# Comparative study of the effect of preoperative hookwire and methylene blue localization techniques on post-operative hospital stay and complications in thoracoscopic pulmonary nodule surgery

**DOI:** 10.1186/s12890-022-02129-1

**Published:** 2022-09-05

**Authors:** Senlin Chu, Ning Wei, Dong Lu, Jie Chai, Shun Liu, Weifu Lv

**Affiliations:** Department of Interventional Radiology, The First Affiliated Hospital of University of Science and Technology of China, Division of Life Sciences and Medicine, University of Science and Technology of China, 17 Lujiang Road, Hefei City, 230001 Anhui Province China

**Keywords:** Patient outcomes, Thoracoscopic surgery, Lung cancer, Hookwire, Methylene blue, Interventional radiology

## Abstract

**Background:**

Direct localization of small and deep pulmonary nodules before thoracoscopic surgery using the hookwire or methylene blue techniques has been recently attempted for better surgical outcomes. In this study, we compare the outcomes of the above two techniques.

**Methods:**

Two hundred and nineteen patients undergoing 135 hookwire and 151 methylene blue techniques in our University Hospital between July 2020 and January 2022 were compared for localization and hospitalization durations, and the complication risk. Other confounders included patients’ age, gender, localization position, nodules location, count, diameter, and depth.

**Results:**

After adjustment of all predictors, the methylene blue technique was associated with a significant 0.6-min (parameter estimate (PE) = −0.568, *p* value = 0.0173) and an 0.7-day shorter localization and hospitalization time (PE = −0.713, *p* value =  < 0.0001) as compared to using the hookwire technique. The hookwire technique was significantly associated with 5 times the risk of developing a post-localization complication (Adjusted Odds Ratio (Adj OR) = 4.52, 95% CI 1.53–13.33) and 3.6 times the risk of developing a pneumothorax (Adj OR = 3.57, 95% CI 1.1–11.62) as compared to adopting the methylene blue technique.

**Conclusions:**

Compared to the hook wire technique, the methylene blue technique offers a shorter procedure and hospitalization stay, as well as a safer post-operative experience.

**Supplementary Information:**

The online version contains supplementary material available at 10.1186/s12890-022-02129-1.

## Introduction

Worldwide, lung cancer remains to be the leading cause of cancer-related morbidity and mortality, accounting for approximately 2.1 million new cancer cases and 1.8 million cancer deaths in 2018 alone [1]. Additionally, it has been the leading cause of cancer and cancer-related deaths in China since 2012 [2, 3]. With the recent advances in diagnostic and treatment modalities for lung cancer, some medical authorities recommended lung screening in the form of low-dose computed tomography (CT) as the most effective modality to reduce mortality from lung cancer by detecting early pulmonary nodules of indeterminate potential [4, 5]. Several studies have demonstrated that the number, size, and nature of the pulmonary nodules predicted the malignant potential of such nodules [6–8].

With the recent recommendations involving lung cancer screening, it is expected that cases with pulmonary nodules will increase in the coming years. Although standard operating procedures may vary according to institution-based recommendations, transbronchial biopsy is frequently used to diagnose central nodules whereas percutaneous biopsy has a high diagnostic efficacy when used to access more peripheral nodules [9]. Pulmonary nodules that were hard to access using either procedure were reserved for thoracoscopic resection, especially those that were multiple and small or those that were located deeper beneath the visceral pleura [10]. Previous research has shown that visual identification of pulmonary nodules during thoracoscopy was minimal when nodules were less than 15 mm in diameter or more than 10 mm below the lung surface [11]. Accordingly, one way to overcome this obstacle was to search for preoperative methods that would let surgeons visually identify pulmonary nodules during thoracoscopy.

The hardships related to finding small or deep pulmonary nodules during thoracoscopy have resulted in the creation of a multitude of localization techniques [12, 13]. Among those techniques, the hookwire implantation and the methylene blue injection techniques have attracted attention as the most widely used but controversial localization techniques in recent years [14, 15]. To our knowledge, those two techniques have never been compared before, and therefore, this study aims at filling that void by comparing the hookwire and methylene blue techniques with regard to their complication rates. We hypothesize that the patient experience as well as the complication rates with the methylene blue technique will be significantly fewer than those with the hookwire implantation technique.

An earlier report comparing the outcomes of pulmonary nodule localization using the hookwire versus the medical adhesive injection techniques has demonstrated that the latter outperformed the former by providing less risk of pneumothorax, less time between localization and surgery, not to mention similar bleeding rates after removal of the hookwire or the injection needles [16]. Another study has shown that the dislodgement rate with the hookwire technique was nearly 13% and that generally, the numbers of patients with complications were greater in the hookwire group of patients in comparison to the methylene group [17]. In our single-institution study, we hypothesize that the superiority of the methylene blue technique concerning the hookwire technique as regards specific patient outcomes can be further confirmed owing to the smaller guage size of the methylene blue injection needle and the hemostatic and adhesive nature of the methylene blue we employed in our study.

## Materials and methods

### The analytic cohort

This study was approved by the Institutional Review Board (IRB) of the Department of Thoracic Surgery of the First Affiliated University Hospital of the University of Science and Technology of China. Signing an informed consent before enrollment was required by all patients. This retrospective cohort study recruited 219 patients with pulmonary nodules who underwent a total of 286 localization procedures between April 2020 and January 2022. These 286 localization procedures comprised 135 procedures using the hookwire technique and 151 procedures using the methylene blue injection techniques. Inclusion criteria included the presence of pulmonary nodules where the largest diameter was less than or equal to 30 mm and where it was difficult to find during the preoperative evaluation by the chief surgeon. Other inclusion criteria included age greater than 18 years, no anatomical or pathological obstacles in the proposed puncture path, a normal general condition rendering the patient fit for surgery, and a normal coagulation profile. Exclusion criteria included patients with preoperative chest CT 3D reconstruction showing that the pulmonary nodules’ positions are deep and close to the hilum of the lung, or that the surface nodules pulled the visceral pleura to create a depressed pleural surface. The choice of the localization technique was based on the clinical features of the patients. Pulmonary nodules were usually localized 24 h before surgery.

### Instruments

The puncturing instruments used in our study included a GE Lingspeed double-row spiral CT scanner (CT scanning was conducted under calm breathing where layer thickness was 2.5 mm, layer spacing was 2.5 mm, and screw pitch was 1.375:1), a hookwwire disposable needle (Model SS510-10, 20G × 100 mm) for locating pulmonary nodules (Ningbo Shengjiekang Biotechnology Co., LTD), a 21G puncture needle for Methylene blue injection (Jiangsu Jichuan Pharmaceutical Group Co., LTD., 2 ml: 20 mg, State drug approval word H32024827), and methylene blue (Kangpaite, Beijing, China, 0.5 ml each, State Food and Drug Administration license No. 3651753, 2013).

### The hookwire group

In the hookwire implantation group, the localization technique followed a specific sequence of steps. First, an initial plain CT of the chest was performed to check the location of the focus field with marker needles for further reference. Second, a high-resolution narrow-focused CT scan of the chest was performed to mark the puncture point (perpendicular to the body surface), estimate the distance from the puncture point to the pulmonary nodule, and choose the best path to the nodule away from any anatomical obstacles such as blood vessels and other structures. Third, the hookwire puncture needle was introduced after local disinfection and analgesia under CT guidance to be positioned within 1 cm around and less than 1 cm deep to the targeted nodule (so as not to obstruct the linear cutting path). Once the hookwire needle was in its desired position, the push rod made to release the anchor hook was withdrawn, the needle was pulled out through the external trocar of the puncture needle, and the distal end of the needle close to the chest wall was cut, and the puncture point was covered with a sterile dressing, and then the patient was sent to the operating room.

### The methylene blue injection group

As for the methylene blue injection group, the procedure was carried out initially in the same manner as regards CT localization of the pulmonary nodule, disinfection of the overlying body surface, and local analgesia. Under CT guidance, a 21G puncture needle was inserted into the lung perpendicular to the pleural surface so that the depth of the needle was estimated at 0.5–1 cm away from the circumference of the pulmonary nodule. After the location of the puncture needle was satisfied, the needle core was withdrawn to confirm that no blood was withdrawn back from the syringe, and then a 1 ml syringe was used to inject a mixture of 0.2 ml of adhesive material and 0.2 ml of methylene blue (ratio of 1:1) at a stable constant speed. After injection, the needle was withdrawn and the CT was rechecked, and the new high-density shadow could be seen in the CT lung window in place of the original pulmonary nodule. It was determined that the localization was satisfactory based on the presence of the aforementioned new high-density shadow. The puncture point was covered with a sterile dressing, and the patient was sent to the operating room.

### Declaration of Helsinki

All methods were carried out in accordance with relevant guidelines and regulations.

### Statistical analysis

Medical records were sought to collect data on each patient’s age, gender, final diagnosis, nodule location, size, and number, the distance of the nodule from the surface of the visceral pleura, the localization date and technique, the position of the patient during localization, complications if applicable, the localization time (defined as the duration of the localization procedure, in minutes), and the surgical time (defined as the durationof the surgical procedure of thoracoscopic resection of the pulmonary nodules). A two-tailed Fisher exact test was used to compare the complication rates between the two groups. Multivariable logistic regression analysis was used to evaluate how the patients’ age, gender, localization technique, localization time, nodule size, nodule distance from the pleural surface, the position of the patient during localization, localization time, and surgical time affected the probability of having a complication of the localization of pulmonary nodules. A multivariable least squares regression analysis was conducted to test the effects of the aforementioned independent variable on both the durations of the localization and the surgical procedures. JMP Pro 16 software (SAS Institute, Cary, NC) was used for the statistical analyses. Statistical significance was defined as *p* value < 0.05 (two-sided).

## Results

A total of 219 patients with pulmonary nodules, undergoing 135 hookwire and 151 methylene blue localizing procedures, constituted our final analytic sample. In our sample (Table 1), there were no significant differences between the hookwire and the methylene blue localization groups as regards age (*p* value = 0.8075), gender proportions (*p* value = 0.5860), the proportions of patients with specific nodule locations (*p* value = 0.3626), the proportions of patients with certain nodule counts (*p* value = 0.4834), median nodule diameter (*p* value = 0.0561), the median distance between nodules and the visceral pleural surface (*p* value = 0.1364), and the median time interval between the end of the localization procedure and the start of surgery for removal of the pulmonary nodules (0.7142).

**Table 1:** Baseline characteristics of 219 patients with pulmonary nodules undergoing 135 hookwire and 151 methylene blue localizing procedures

Variable	Hookwire technique 107 patients (48.86%)	Methylene blue technique 112 patients (51.14%)	*p* value
	N (%)	N (%)	
Age (years)^#^	54 (10.86)	54.46 (11.06)	0.8075
Gender			0.5860
Male	43 (40.19%)	41 (36.6%)	
Female	64 (59.81%)	71 (63.4%)	
Patient position			< 0.0001*
Supine	39 (36.45%)	15 (11.36%)	
Prone	30 (28.04%)	29 (21.97%)	
Right lying	21 (19.63%)	32 (24.24%)	
Left lying	17 (15.88%)	56 (42.43%)	
Nodule location			0.3626
Right upper lobe	51 (37.78%)	49 (32.45%)	
Right middle lobe	6 (4.44%)	13 (8.61%)	
Right lower lobe	23 (17.04%)	35 (23.18%)	
Left upper lobe	34 (25.19%)	35 (23.18%)	
Left lower lobe	44 (8.32%)	19 (12.58%)	
Nodule count			0.4834
One	83 (77.57%)	81 (72.32%)	
Two	20 (18.69%)	23 (20.54%)	
Three	4 (3.74%)	8 (7.14%)	
Complications			0.0126*
None	115 (85.19%)	140 (92.72%)	
Pneumothorax	13 (9.63%)	11 (7.28%)	
Hemoptysis	7 (5.18%)	0 (0%)	
Nodule diameter (mm)^#^	8 (4)	9 (6)	0.0561
Distance between nodule and pleural surface (mm)^#^	9 (11)	10 (12)	0.1364
Localization time (min)^#^	8 (3)	7 (3)	0.0007*
Localization-operation interval (hours)^#^	17 (3.5)	17 (6)	0.7142
Duration of surgery (min)^#^	114 (92)	95 (61.5)	0.0446*
Hospitalization time (days)^#^	6 (3)	5 (2)	< 0.0001*

*N* number, % percentage, *IQR* Interquartile range, *mm* millimeter, *min* minutes

^#^For continuous variables, summary statistics have been calculated in the form of either the mean and standard deviation for normally distributed variables (such as age), or the median and interquartile ranges for non-normally distributed variables (such as nodular diameter, nodular distance from the pleura, localization time, localization-operation interval, duration of surgery, and hospitalization time

For categorical variables (such as gender, patient position, nodule location, nodule number, and complications), the summary statistics were calculated in the form of the frequency and percentage of the total observations, whether these were patients or localization procedures

^*^Statistically significant

On the other hand, the proportion of patients assuming the supine position was significantly larger in the hookwire group (36.45%) than in the methylene blue group (11.36%) (*p* value =  < 0.0001), and the proportion of patients assuming the left lying position was significantly higher in the methylene blue group (42.43%) than in the hookwire group (15.88%) (*p* value =  < 0.0001). Also, the proportion of patients who presented with hemoptysis was 5.18% in the hookwire group as compared to none in the methylene blue group (*p* value = 0.0126). As regards the median localization time, duration of surgery, and hospitalization time, they were all significantly shorter in the methylene blue group than in the hookwire group (*p* values = 0.0007, 0.0446, and < 0.0001, respectively; Table1).

Using multivariable linear regression to quantify the effect of the localization technique (among other variables) on the duration of the localization procedure (Table 2), we found that, after adjustment of other predictors, the use of the methylene blue localization technique was significantly associated—on average—with a reduction in the localization time by nearly 0.6 min (parameter estimate (PE) = − 0.568, *p* value = 0.0173). Also, having a pulmonary nodule located in the lower lobe of the right lung was significantly associated—on average—with an increase in the localization time by approximately 1.3 min (PE = 1.344, *p* value = 0.016).

**Table 2:** Multivariable linear regression analysis of the association between age, nodule diameter, nodule depth, gender, patient position, nodule location, nodule count, and localization technique and the duration of the localization procedures (in minutes), and hospitalization (in days) among our study participants

Variable	Duration of localization (minutes)	Duration of hospitalization (days)
	Adjusted PE (*p* value)	Adjusted PE (*p* value)
Age (years)	− 0.014 (0.4914)	0.044 (0.0007*)
Nodule diameter (mm)	− 0.028 (0.6785)	− 0.063 (0.1308)
Nodule depth from surface (mm)	0.004 (0.897)	0.038 (0.0421*)
Gender		
Male	0.444 (0.0526)	− 0.208 (0.1452)
Female	− 0.444 (0.0526)	0.208 (0.1452)
Patient position		
Supine	1.199 (0.0049*)	− 0.081 (0.759)
Prone	− 0.689 (0.1202)	0.136 (0.6269)
Right lying	− 0.415 (0.4794)	0.102 (0.7826)
Left lying	− 0.094 (0.8414)	− 0.157 (0.591)
Nodule location		
Right upper lobe	− 0.387 (0.3668)	0.332 (0.2153)
Right middle lobe	− 0.796 (0.3333)	0.249 (0.6273)
Right lower lobe	1.344 (0.016*)	0.045 (0.8958)
Left upper lobe	− 0.017 (0.9775)	0.101 (0.7909)
Left lower lobe	− 0.144 (0.8099)	− 0.727 (0.0555)
Nodule count		
One	− 0.294 (0.476)	− 0.389 (0.131)
Two	− 0.038 (0.934)	0.432 (0.1302)
Three	0.332 (0.6144)	− 0.043 (0.916)
Localization technique		
Hookwire	0.568 (0.0173*)	0.714 (< 0.0001*)
Methylene blue	− 0.568 (0.0173*)	− 0.714 (< 0.0001*)

*PE* Parameter Estimate, *mm* millimeter

*Statistically significant

As regards the adjusted effect of the localization technique on the hospitalization time (in days), we found that the use of the methylene blue technique was significantly associated with a reduction in hospital stay by an average of 0.7 days in comparison to using the hookwire technique (PE = − 0.713, *p* value =  < 0.0001). Moreover, Table 2 also shows that with every one-year increase in the patient’s age there is a corresponding 0.04-day increase in hospital stay after any localization technique (PE = 0.044, *p* value = 0.0007). Also, our analysis showed that every 1 mm increase in the depth of the pulmonary nodule was significantly associated with a 0.04-day increase in hospital stay after any localization technique (PE = 0.038, *p* value = 0.0421).

Employing a multivariable logistic regression analysis, the presence of a pulmonary nodule in the upper lobe of the left lung was significantly associated with five times the risk of developing a post-localization complication as compared to pulmonary nodules situated in the upper lobe of the right lung, regardless of other predictors (Adj OR = 5.36, 95% CI 1.15–25.04). On the other hand, data in Additional file 1: tables S1 and S2 (not published) showed that the localization technique had no significant effects on either the duration of surgery (*p* value = 0.0787) or the time interval between the end of the localization procedure and the beginning of surgery (*p* value = 0.8708), respectively.

To quantify the effect of our included predictors on the probability of developing a complication after the localization procedure, we found that, after adjustment of other predicting factors, the localization technique, the nodule count, and the depth of the nodule from the visceral pleural surface were the most significant factors affecting the probability of complications after localization (Table 3). After adjustment of other predictors, we found that adopting the hookwire technique was associated with approximately 5 times the risk of developing a complication as adopting the methylene blue technique (Adjusted Odds Ratio (Adj OR) = 4.52, 95% CI 1.53–13.33). We also found that adopting the hookwire localization technique was associated with 3.6 times the odds of developing pneumothorax after the localization procedure as using the methylene blue technique (Adj OR = 3.57, 95% CI 1.1–11.62).

**Table 3:** Multivariable logistic regression analysis of the association between age nodule diameter, nodule depth, gender, patient position, nodule location, nodule count, and localization technique and the probability of developing complications or pneumothorax among our study participants

Variable	Probability of complications	Probability of pneumothorax
	Adjusted OR (95% CI)	Adjusted OR (95% CI)
Age (years)	0.99 (0.95–1.04)	1.004 (0.95–1.06)
Nodule diameter (mm)	0.97 (0.83–1.12)	0.96 (0.81–1.14)
Nodule depth from surface (mm)	1.07 (1.01–1.13)*	1.04 (0.97–1.11)
Gender		
Male	Reference	Reference
Female	1.02 (0.39–2.65)	0.67 (0.22–2.08)
Patient Position		
Supine	Reference	Reference
Prone	2.27 (0.54–9.62)	8.26 (1.09–62.7)*
Right lying	0.48 (0.101–2.31)	2.1 (0.26–16.64)
Left lying	2.21 (0.51–9.53)	9.2 (1.07–78.83)*
Nodule location		
Right upper lobe	Reference	Reference
Right middle lobe	1.63 (0.15–17.32)	1.88 × 10^–8^ (0-N/A)
Right lower lobe	1.06 (0.23–4.97)	1.35 (0.27–6.78)
Left upper lobe	5.36 (1.15–25.04)*	5.43 (0.66–44.59)
Left lower lobe	2.75 (0.52–14.56)	1.84 (0.24–14.37)
Nodule count		
One	Reference	Reference
Two	2.62 (0.87–7.84)	5.82 (1.69–20.1)*
Three	11.31 (2.23–57.37)*	17.79 (2.98–106.13)*
Localization technique		
Methylene blue	Reference	Reference
Hookwire	4.52 (1.53–13.33)*	3.57 (1.1–11.62)*

*OR* Odds Ratio, *CI* Confidence Interval, *N/A* Not applicable, *mm* millimeter

*Statistically significant

Patients lying prone or on their right side during localization were significantly associated with eight (Adjusted OR = 8.26, 95% CI 1.09–62.7) and nine (Adjusted OR = 9.2, 95% CI 1.07–78.83) times the risk of developing pneumothorax, regardless of the localization technique, as those lying supine, respectively. Patients who had two or three pulmonary nodules during localization were significantly associated with nearly 6 (Adjusted OR = 5.82, 95% CI 1.69–20.1) and 18 (Adjusted OR = 17.79, 95% CI 2.98–106.13) times the risk of developing pneumothorax as those who only present with one nodule. Finally, Additional file 1: Table S3 (not published) showed that the localization technique was not significantly associated with an increased risk of developing hemoptysis (*p* value = 0.9977).

## Discussion

In this study, we have shown that both the hookwire and the methylene blue techniques are useful in localizing pulmonary nodules before thoracic surgeries aiming at wedge or segmental resection of such nodules. However, we have also exhibited how the methylene blue technique was superior to the hookwire technique in terms of significantly reducing the duration of the localization procedures, the duration of surgery, as well as the duration of hospitalization for patients with pulmonary nodules. Moreover, the use of the methylene blue technique was associated with a significantly lower probability of developing pneumothorax. For those reasons, we agree with the conclusion that the methylene blue technique should be chosen over the hookwire technique for the preoperative localization of pulmonary nodules.

We realize that although the difference in localization time between the hookwire and the methylene blue groups was statistically significant, it was not meaningfully significant within the context of the overall surgical procedure of thoracoscopic resection of pulmonary nodules. Yet, this difference may be meaningful in other clinical contexts, such as an outpatient interventional radiology (IR) clinic, where the localization of the nodules is not performed in continuity with the surgical procedure. That is why we conclude that our results may not be generalizable to all patients but only to those who are undergoing localization in the IR outpatient clinics and not necessarily in the surgical operating room.

This is not the first study to investigate the effectiveness of both localization techniques [18, 19], not to mention compare their effectiveness among a common patient population [17]. The most distinguishing feature of our study, as compared to the outlined studies, was the lower probability of developing a complication after using methylene blue as compared to the hookwire technique. It is well known among surgeons that methylene blue is a non-toxic dye that rapidly polymerizes into blue colloids in water or tissue fluids and can “glue” the broken ends of blood vessels, promoting vasoconstriction and blood coagulation, and therefore is considered biologically safe [20]. That was the reason why methylene blue diffusion complications did not occur on the pleural surface in patients of the methylene blue group.

On the other hand, the use of the hookwire technique was shown in previous studies to exhibit a high rate of dislodgement (4–20%), with the unfavorable consequences of increasing the incidences of pneumothorax and peri-lesional hemorrhage [21]. Because we were more patient-focused than technique-focused, we only included patients with successful localization techniques as our final analytic cohorts. With respect to the “dislodgement of the hookwire”, 15 (14%) out of 107 patients undergoing the hookwire technique in our study showed dislodgement, which was immediately managed by the responsible hospital staff so that the localization procedure was completed and the patients were sent forward to surgery. Furthermore, the hookwire dislodgement rate is unique to that group and cannot be used in comparison to the methylene blue group because this measure will equal 0 in the methylene blue group, creating a false sense of superiority of the methylene blue technique.

In our study, we have shown that the hookwire technique was associated with 3.6 times the risk of post-procedure pneumothorax as the methylene blue technique. In congruence with other reports, multiple puncture sites (due to multiple nodules) were among the most contributing factors to the development of pneumothorax in our patient cohort [22]. On the other hand, the use of the methylene blue technique was shown to simultaneously localize multiple nodules while yielding less risk of developing pneumothorax in other studies [23]. Still, apart from previous pathological lung conditions that may co-exist during the localization procedure and influence non-procedure related complications, the number of pulmonary nodules is a powerful predictor of post-procedure pneumothorax even when using the methylene blue technique. More studies are warranted to compare the two localization procedures in the context of various pathological lung conditions to assess their true effectiveness.

As regards hemoptysis, seven patients in the hookwire group in our study experienced hemoptysis versus none from the methylene blue group. Although the sample size of patients with post-procedure hemoptysis were too small to predict a causal relationship between the hookwire technique and the development of hemoptysis, the association between the hookwire technique and the post-operative bleeding has been shown in previous studies [17, 20, 22]. Our inclusion criteria specified participants with a normal general condition and coagulation profile who exhibited no additional lung pathologies, and therefore, there was no reason for us to believe that the cause of hemoptysis in our patient cohort was due to a preoperative condition. Also, the fact that the methylene blue used in our study was mixed with an adhesive glue that rendered it polimerizable seconds after injection reduces the possibility of an intraoperative cause of hemoptysis. Yet, there were studies outlining other patient- and procedure-specific factors that may have been responsible for the development of hemoptysis or pulmonary hemorrage in general, such as the number of nodules, pre-existing medications, repositioning of the patients, etc. [17, 20, 23]. Therefore, we warrant further studies to differentiate between patient-related and procedure-related predictors of pulmonary hemorrhage in patients undergoing localization techniques for pulmonary nodules.

Unlike other studies, we chose to ignore cough and post-operative pain as significant complications after localization procedures, unless these symptoms were supported by additional investigations suggesting a more hazardous diagnosis such as pneumothorax, acute myocardial infarction, pulmonary embolus, or air embolism. It is a well-known fact that chest pain and cough can be the initial symptoms of pulmonary embolism. Also, air emboli can result from either localization techniques, causing dyspnea, chest pain, irritating cough, or ultimately the death of a patient. Unfortunately, multiple factors could be responsible for air emboli or pneumothorax during localization techniques, including the hookwire needle, the resection procedure itself, or other pre-operative or intra-operative lung manipulations. Therefore, we did not find any studies confirming the source of air emboli during localization techniques. Future studies investigating the occurrence of such a complication at different stages of the localization processes are greatly warranted.

Despite the advantages of the methylene blue technique that we have demonstrated througout our analysis, the following precautions need to be followed during daily use. For subpleural pulmonary nodules (vertical distance from the pleura < 3 cm), the tip of the injector needle should be kept at 1–1.5 cm away from the nodules to avoid injecting the methylene blue into nodule which might affect the pathological diagnosis. The injector needle should also be used superficially to allow for easy palpation of the nodules during the operation. We recommend that the relative amounts of methylene blue and the adhesive material be in the ratio of 1:1 (0.2 ml each), which will significantly reduce the incidence of irritating cough. Additionally, the glue block can be formed quickly to avoid leakage to the subpleura. Immediately after the injection of the liquid, the polymerization produces heat and burning pain, which can also reduce the incidence of pleural reactions.

The major strength of our study is the novelty of the idea and the innovation in the adhesive substance which combines methylene blue and an adhesive liquid. Previous studies comparing the two localization methods either used a medical adhesive without methylene blue [20] or used methylene blue without an adhesive substance [17]. The current study is limited by the relatively small sample size of the study participants, which was reflected in the wide confidence intervals in our significant statistical results. Future studies with larger sample sizes are required to confirm the results obtained from this analysis.

## Conclusion

The results from our analysis suggest that the hookwire and the methylene blue localization techniques are not similar methods for preoperative pulmonary nodule localization for patients suspected of having lung cancer. The methylene blue technique is superior to the hookwire technique in terms of providing fewer complications for patients as well as shortening the time for their localization procedures as well as their hospital stays. However, one advantage of the hookwire technique that is not present in the methylene blue technique is that it can mark not only the localization of the nodule from the pleural surface but also the depth of the nodule as well. Accordingly, it is reasonable to conclude that the methylene blue technique is more advisable for preoperative peripheral pulmonary nodule localization owing to its better patient outcomes after surgery. In order for our results to be more meaningful, it is reasonable to say that they are not generalizable for all patients, and is more actionable for patients doing localization of pulmonary nodules within IR units.

## Supplementary Information

**Additional file 1. Supplementary Table S1**.Multivariable Linear Regression Analysis of the Association Between Age, Nodule Diameter, Nodule Depth, Gender, Patient Position, Nodule Location, Nodule Count, and Localization Technique and the Duration of Surgery (in minutes) Among Our Study Participants.** Supplementary Table S2**.Multivariable Linear Regression Analysis of the Association Between Age, Nodule Diameter, Nodule Depth, Gender, Patient Position, Nodule Location, Nodule Count, and Localization Technique and the Duration of the Interval Between the End of Localization and the Start of Surgery (in hours) Among Our Study Participants.** Supplemental Table S3**.Odds Ratios for The Effect of the Localization Technique on the Risk of Hemoptysis.

## Data Availability

The datasets used and/or analyzed during the current study available from the corresponding author on reasonable request.
