# Supplementary material for: Comparative study of the effect of preoperative hookwire and methylene blue localization techniques on post-operative hospital stay and complications in thoracoscopic pulmonary nodule surgery
Source: BMC Pulm Med. 2022 Sep 5;22:336. doi: 10.1186/s12890-022-02129-1 (PMC9446788; doi:10.1186/s12890-022-02129-1)
Supplement: Supplementary file 1 — Additional file 1. Supplementary Table S1.Multivariable Linear Regression Analysis of the Association Between Age, Nodule Diameter, Nodule Depth, Gender, Patient Position, Nodule Location, Nodule Count, and Localization Technique and the Duration of Surgery (in minutes) Among Our Study Participants. Supplementary Table S2.Multivariable Linear Regression Analysis of the Association Between Age, Nodule Diameter, Nodule Depth, Gender, Patient Position, Nodule Location, Nodule Count, and Localization Technique and the Duration of the Interval Between the End of Localization and the Start of Surgery (in hours) Among Our Study Participants. Supplemental Table S3.Odds Ratios for The Effect of the Localization Technique on the Risk of Hemoptysis. [file 12890_2022_2129_MOESM1_ESM.docx]

**Supplementary Table S1**

*Multivariable Linear Regression Analysis of the Association Between Age, Nodule Diameter, Nodule Depth, Gender, Patient Position, Nodule Location, Nodule Count, and Localization Technique and the Duration of Surgery (in minutes) Among Our Study Participants*

| Term | Estimate | Std Error | t Ratio | Prob>\|t\| |
| --- | --- | --- | --- | --- |
| Intercept | 49.7058 | 19.48649 | 2.550782 | 0.011489 |
| Age | 0.642531 | 0.316546 | 2.029816 | 0.043687 |
| Nodule diameter (mm) | 2.026771 | 1.024476 | 1.978349 | 0.049248 |
| Distance from nodule to pleura (mm) | 0.761564 | 0.454549 | 1.675429 | 0.095397 |
| Gender[female] | 1.385328 | 3.510944 | 0.394574 | 0.693573 |
| Gender[male] | -1.38533 | 3.510944 | -0.39457 | 0.693573 |
| position 2[Left lying] | -9.05655 | 7.213866 | -1.25544 | 0.210771 |
| position 2[Prone] | 2.821976 | 6.820563 | 0.413745 | 0.6795 |
| position 2[Right lying] | -0.73687 | 9.034584 | -0.08156 | 0.935077 |
| position 2[Supine] | 6.971445 | 6.498085 | 1.072846 | 0.28462 |
| Nodule location 2[Lower left] | -12.8326 | 9.211321 | -1.39313 | 0.165111 |
| Nodule location 2[Lower right] | -18.5497 | 8.537093 | -2.17284 | 0.030956 |
| Nodule location 2[Middle right] | 13.66082 | 12.65782 | 1.079239 | 0.281768 |
| Nodule location 2[Upper left] | 14.71736 | 9.284512 | 1.585152 | 0.114496 |
| Nodule location 2[Upper right] | 3.004162 | 6.608781 | 0.454571 | 0.649906 |
| Nodule count[1] | -13.4696 | 6.347573 | -2.122 | 0.035056 |
| Nodule count[2] | 15.85955 | 7.040776 | 2.252528 | 0.025364 |
| Nodule count[3] | -2.38999 | 10.14103 | -0.23568 | 0.813923 |
| Localization technique[Hookwire] | 6.457594 | 3.654638 | 1.766959 | 0.078745 |
| Localization technique[Methylene Blue] | -6.45759 | 3.654638 | -1.76696 | 0.078745 |
|  |  |  |  |  |

mm= millimeters

**Supplementary Table S2**

*Multivariable Linear Regression Analysis of the Association Between Age, Nodule Diameter, Nodule Depth, Gender, Patient Position, Nodule Location, Nodule Count, and Localization Technique and the Duration of the Interval Between the End of Localization and the Start of Surgery (in hours) Among Our Study Participants*

| Term | Estimate | Std Error | t Ratio | Prob>\|t\| |
| --- | --- | --- | --- | --- |
| Intercept | 16.4111 | 2.325918 | 7.055751 | 2.69E-11 |
| Age | 0.009171 | 0.037783 | 0.242737 | 0.808455 |
| Nodule diameter mm | -0.11488 | 0.122282 | -0.93945 | 0.348621 |
| Distance from nodule to pleura mm | -0.05351 | 0.054255 | -0.98625 | 0.325188 |
| Gender[female] | -0.89163 | 0.419068 | -2.12764 | 0.034579 |
| Gender[male] | 0.891626 | 0.419068 | 2.12764 | 0.034579 |
| position 2[Left lying] | 0.98906 | 0.861051 | 1.148667 | 0.252052 |
| position 2[Prone] | -0.56519 | 0.814106 | -0.69425 | 0.488326 |
| position 2[Right lying] | 0.432639 | 1.078372 | 0.401196 | 0.6887 |
| position 2[Supine] | -0.85651 | 0.775615 | -1.1043 | 0.270777 |
| Nodule location 2[Lower left] | 0.839941 | 1.099468 | 0.763952 | 0.445787 |
| Nodule location 2[Lower right] | -1.57941 | 1.018992 | -1.54998 | 0.122713 |
| Nodule location 2[Middle right] | 0.642481 | 1.510843 | 0.425247 | 0.671109 |
| Nodule location 2[Upper left] | 0.045367 | 1.108204 | 0.040937 | 0.967386 |
| Nodule location 2[Upper right] | 0.051623 | 0.788827 | 0.065443 | 0.947886 |
| Nodule count[1] | 1.605184 | 0.757649 | 2.118637 | 0.035343 |
| Nodule count[2] | 0.631432 | 0.840391 | 0.751355 | 0.453313 |
| Nodule count[3] | -2.23662 | 1.210439 | -1.84777 | 0.066097 |
| Localization technique[Hookwire] | -0.07106 | 0.436219 | -0.1629 | 0.870756 |
| Localization technique[Methylene Blue] | 0.071062 | 0.436219 | 0.162905 | 0.870756 |

mm= millimeter

**Supplemental Table S3**

*Odds Ratios for The Effect of the Localization Technique on the Risk of Hemoptysis*

| Level1 | /Level2 | Odds Ratio | Prob>Chisq | Lower 95% | Upper 95% |
| --- | --- | --- | --- | --- | --- |
| Methylene Blue | Hookwire | 1.55242E-17 | 0.997708161 | 0 |  |
| Hookwire | Methylene Blue | 6.44157E+16 | 0.997708161 | 0 |  |
